# Stakeholder engagement does not guarantee impact: A co-productionist perspective on model-based drought research

**DOI:** 10.1177/03063127231199220

**Published:** 2023-09-27

**Authors:** Catharina Landström, Eric Sarmiento, Sarah J Whatmore

**Affiliations:** 1 Chalmers University of Technology, Gothenburg, Sweden; 2 University of Oxford, Oxford, UK; 3 Texas State University, San Marcos, TX, USA

**Keywords:** computer modelling, stakeholder engagement, data, drought governance space, water management

## Abstract

Stakeholder engagement has become a watchword for environmental scientists to assert the societal relevance of their projects to funding agencies. In water research based on computer simulation modelling, stakeholder engagement has attracted interest as a means to overcome low uptake of new tools for water management. An increasingly accepted view is that more and better stakeholder involvement in research projects will lead to increased adoption of the modelling tools created by scientists in water management. However, we cast doubt on this view by drawing attention to how the freedom of stakeholder organizations to adopt new scientific modelling tools in their regular practices is circumscribed by the societal context. We use a modified concept of co-production in an analysis of a case of scientific research on drought in the UK to show how relationships between actors in the drought governance space influence the uptake of scientific modelling tools. The analysis suggests an explanation of why stakeholder engagement with one scientific project led to one output (data) getting adopted by stakeholders while another output (modelling tools) attracted no discernible interest. Our main objective is to improve the understanding of the limitations to stakeholder engagement as a means of increasing societal uptake of scientific research outputs.

Computer simulation modelling in environmental science can struggle to demonstrate its practical utility. As ‘demands for scientists to generate *impactful* research are ubiquitous’ (McLellan, 2021, p. 100), this is a cause for concern among environmental scientists and funding agencies both. Stakeholder involvement in research projects has become a key approach to overcoming this challenge. Articles offering advice on how to improve engagement with stakeholders in environmental modelling projects abound. For example: ‘Stakeholder involvement can serve to increase the quality of decision support systems (DSSs) and increase the perceived legitimacy of DSS outputs’ (Sandink et al., 2016, p 193). Like other environmental scientists discussing the issue, Sandink et al. (2016) insist that technology transfer fails because of inadequate engagement with stakeholders and end-user communities in the development and design of model-based tools. In another paper aimed at environmental modellers, Parrott (2017) offers practical advice for how environmental modelling projects can open up the research process to involvement of relevant stakeholders and thereby create knowledge that will help to address wicked problems. Wu et al. (2016) argue that inviting stakeholders to provide input to the scientific development of optimization algorithms, to identify solutions to real world problems, will improve the applicability of the research outputs and thus the uptake. Malard et al. (2017) identify the complexity of most modelling approaches as a barrier to effective stakeholder involvement and advocate for combining system dynamics modelling (which they think is easier for the non-scientist to comprehend) with physical models by using their new coupling software. In a similar vein, Allison et al. (2018) encourage modellers to consider the need for simplicity when creating models for use by stakeholders and present their own project developing a decision support system as an example of how to accomplish this. These examples clearly share a general stance, despite awareness of the repeated failure of this approach—the quality of actual stakeholder involvement is identified as the problem, not the strategy of trying to achieve impact in this way.

In our opinion, a science and technology studies (STS) perspective should suggest caution about stakeholder engagement as a way to promote uptake of computer modelling tools in environmental management. It might appear that STS discussions of participatory research would support the expectations of the environmental scientists by being positive to stakeholder engagement and critical of perfunctory attempts to manufacture consent by inviting people to events staged to silence opposition (Chilvers & Kearnes, 2020; Smallman, 2020). However, the environmental scientists and the STS scholars do not talk about the same stakeholders.

Environmental scientists regularly use the term ‘stakeholder’ with reference to organizations with statutory or economic interests in the issues studied (Giordano et al., 2020). For example, water scientists in the UK consider government agencies and water utility companies to be key stakeholders with which to engage (e.g. Goodwin et al., 2019). As a consequence of this definition, the individuals who collaborate with scientists in water research projects are usually professionals, with backgrounds in natural science or engineering, representing stakeholder organizations. The scientists expect that these representatives will acquire in-depth understanding of the scientific knowledge and modelling tools developed in the projects and bring these into regular use in their own organizations. In contrast, STS discussions about stakeholder participation focus on lay publics that are in some way affected by the issue and the scientific research on it (Landström, 2020). For the STS scholar, the purpose of involving lay publics with environmental (and other) science is always to some extent about democratizing access to scientific knowledge and empowering marginalized publics (Chilvers & Kearnes, 2020). STS scholars have even identified the alliance of scientists and institutional and corporate stakeholders as causing the problem that public participation is intended to counter (Wynne, 2006).

Our caution against relying on the involvement of professionals representing stakeholder organizations to increase the uptake of model-based tools in environmental management is not based on discussions of participatory research but draws on analyses of institutional relationships. Case studies of how outputs from scientific research become tools and knowledge used in public and private organizations identify various institutional factors as critical (Oliver & Boaz, 2019). We argue that although engagement with formal stakeholder organizations can promote communication, in both directions, it is not likely to increase the uptake of model-based tools by the stakeholder organizations: Relationships between organizations involved with the environmental system act as constraints.

Our argument is developed through an empirical study of a research programme on drought in the UK that embraced stakeholder involvement from the outset. The case study focuses on the natural science modellers in one of the projects within the drought programme. The ethnography captures how the scientists interpreted what stakeholder engagement involved and how they attempted to create outputs of interest to stakeholder organizations. To analyse the interplay of direct interaction of scientists and stakeholder representatives and the relations between stakeholder organizations we draw on the well-established concept of co-production (Jasanoff, 2004a).

## Conceptual tools for capturing entanglements

The notion of co-production has proved fruitful as a way to articulate and detail the intertwining of scientific knowledge production with other areas of society. The concept effectively captures various connections between science as institutional practice and other organized activities in society. Jasanoff (2004a) presents co-production as an idiom, a vocabulary for ‘interpreting and accounting for complex phenomena so as to avoid the strategic deletions and omissions of most other approaches in the social sciences’ (p. 3). We follow this in formulating an argument against the omission of societal context in discussions about the potential of stakeholder engagement to increase the uptake of research outputs from water science in water management practice.

The idiom distinguishes four aspects of co-production: ‘making *identities*, making *institutions*, making *discourses* and making *representations*’ (Jasanoff, 2004b, p. 38). Here, making identities concern the creation of shared knowledge and values that define people (and things) as belonging to a particular field. Institutions offer established routes for accreditation of knowledge, routines for how to validate new knowledge. Discourses are about the naming of things; how new phenomena or problems are talked about brings with them implicit models of nature and society. Representations focus on how nature is understood to work, explanations of processes and the mechanisms producing the phenomena observed.

The notion of co-production encourages us to widen the scope of analysis from the face-to-face interaction of scientists and stakeholders in research projects to also consider the influence of the societal order of knowledge and power. In present day UK drought management the institutional aspect of co-production requires further elaboration and to this end we deploy the notion of drought governance space.

Lange and Cook (2015) understand there to be a ‘drought governance space’ in terms of three features:

First, there is a separate body of legal rules in relation to drought that demarcates the boundaries of this governance space. … Secondly, the boundaries of this governance space can be mapped onto groups of staff within DEFRA [Department of Environment, Food and Rural Affairs], the EA [Environment Agency], Nature Conservation organizations, and water companies whose work is concerned with managing drought. … Thirdly, a specific set of policy ideas marks out a distinct governance space for managing drought. (Lange & Cook, 2015, p. 257)

These features outline a domain within which ‘a complex web of relationships that cross national, regional, and local levels’ (Lange & Cook, 2015, p. 255) can be discerned. Further, the concept is distinguished by the explicit recognition of the ‘importance of networks composed of hybrid public–private actors for steering behaviour and steering across different levels of political and legal authority’ (Lange & Cook, 2015, p. 254).

The notion of a drought governance space aligns with a conceptual shift away from understanding environmental decision making as centralized, top-down government, in which national actors make policy that is implemented locally. Betsill and Bulkeley (2006) identify the emergence of multi-level environmental governance as involving a new type of network that links actors across global, regional and local levels. In their study of climate change, environmental governance ‘takes place through processes and institutions operating at and between a variety of scales and involving a range of actors with different levels and forms of authority’ (Betsill & Bulkeley, 2006, p. 141).

With regard to the responsibility for managing and researching droughts in the UK, in the 1990s a top-down organization with strong institutions was dismantled with the privatization of the water sector and a re-purposing of government departments and public agencies. Here the validation and accreditation of new scientific knowledge that in Jasanoff’s idiom of co-production took place in institutions is instead distributed across actors of various types and with different roles and reach, such as water utility companies, regulatory agencies, universities and expert consultancy firms. This was visible from the very beginning of our case study.

## Case study context

The UK Drought and Water Scarcity (DWS) programme (running from 2014 to 2019) was a research endeavour funded by the Natural Environment Research Council (NERC) that comprised five separate projects. Prompted in part by the drought threatening the 2012 Olympic Games in London, the DWS programme brought together expertise from research on drought and scientists with a track record in other areas of water research. It started out with four multi-disciplinary, multi-institutional, multi-sited consortia projects, and after three years a fifth, cross-cutting, project was launched to consolidate the knowledge generated and amplify societal impacts. In addition to creating new scientific knowledge, the projects in the DWS programme were to contribute to the improvement of drought management in the UK. All projects included collaboration with stakeholder organizations and several projects established relationships with other interested actors—connecting with society was emphasized in the concluding impact project.

This paper focuses on one of the four consortia projects. The aim of the project was to understand the impacts on nature and society of droughts in the UK. The project included natural and social scientists, and collaboration occurred mainly along disciplinary lines except for the computer modelling that brought natural scientists together across disciplinary boundaries. The innovative, interdisciplinary modelling makes the project particularly interesting to the present analysis as it was promising to offer a new way to model droughts that would be useful in drought management. Already in the development of the project proposal the lead scientists made contact with stakeholder organizations in the drought governance space that they wanted to engage with, including water utility companies, government regulators and technical consultants.

## Materials and methods

The research underpinning this paper was undertaken by the authors as participant observers in one of the empirical projects in the DWS programme. The ethnographic approach was informed by previous STS case studies of computer-centred environmental research (e.g. Lahsen, 2005; Mayernick, 2019; Sundberg, 2009). As researchers in the project, we had access to documentation generated for internal use and to presentation slides from team meetings. The research included attending project team meetings, stakeholder workshops and public conferences. We also video-recorded meetings attended by scientific modellers and had informal conversations with project scientists about their work (see, e.g. Jaton, 2017; Jensen, 2020).

To generate materials specifically for the purpose of our STS analysis we carried out semi-structured interviews with natural science modellers in the project, following common case study practice (Jensen, 2020; Mauz & Granjou, 2013). We also interviewed drought modellers in the other DWS projects, modellers not connected to the programme, and stakeholder representatives. The interviews were audio recorded, professionally transcribed and analysed with support of NVivo software. The guiding principle in our analysis was to follow the actors (Latour, 1987) and the material was interpreted with the intent of understanding the perspectives of the natural science modellers on the people and things they interacted with in their pursuit of knowledge about drought (Sundberg, 2009).

## Rendering drought digital

The key research technique in the DWS programme overall was computer simulation modelling, today a common approach in environmental science. Water scientists, including hydrologists, had adopted digital technologies early, in the 1970s using punch cards to run models on mainframe computers (Beven, 2019). Computer simulation models offer water scientists a way to quantify causal processes and predict future states of complex systems.

Computer simulation has been studied in STS and cognate fields for decades. A key feature that enables scientists to learn by modelling is identified by Morrison and Morgan (1999) as epistemic autonomy originating in such models’ ability to mediate between theory and data. Sismondo (1999) points out that models are useful for addressing ‘what if’ questions at minimal cost and effort. Ethnographers have examined the role of computer models in scientific research practice, highlighting tensions arising in their wake as field-based science get marginalized (Sundberg, 2006).

Computer simulation has also been an important approach in water management since the PC became common in the offices of water management organizations (Wurbs, 1998). The requirements for water management models differ from those demanded of scientific models. Landström (2023) highlights the importance of the usability of models to professionals with different disciplinary backgrounds and whose practical objectives are locally defined. The notion of fit-for-purpose modelling, including institutional fit features in discussions of model use in environmental management indicating how well a modelling tool reflects the purposes and practices in the organization using it (Hamilton et al., 2022).

The organizations identified as stakeholders with key roles in the UK drought governance space, for example, water utility companies and regulatory agencies, use computer modelling extensively to plan their actions and analyse possible outcomes. Other stakeholders, such as technical consultants, also use models. Some develop modelling software, and such proprietary software becomes a company asset; it can earn revenue through licensing fees and fees charged for training courses attended by consultants and modellers in other organizations.

In the project we studied (hereafter the drought project), the computer modelling was organized as a relay, starting with climate models producing drought data sets that were to be used in two different hydrological modelling approaches, which would provide additional data for water quality and water resources models. With added hydrological and water quality data the event sets would then be used in ecological and economic modelling. The climate modellers used the regional climate model HadRM3P, supplied by the UK MetOffice, to generate data that amounted to what instruments in the physical environment would record if a drought were occurring. Before the modelling relay could begin the modellers had to agree on what to model, that is, what a drought would be in modelling terms.

To most people, drought is a lack of rain and a diminishing amount of water in the environment—free flowing in rivers and lakes as well as decreasing amounts of ground water and reduced soil moisture. We perceive it as the drying out of the ground, dry riverbeds, low water levels in lakes, dying plants and perhaps dust in the air. However, the experience of drought is very different from drought represented in a scientific computer model (Laborde, 2015). The drought project modellers discussed how to define the phenomenon in a way that would work for all involved disciplines and research questions. In an interview a scientist commented on this discussion:

There’s a proposal at the moment to define drought by using hydrological models. We put into the hydrological models long climate records—maybe 100 years of climate data—and out of those models, comes river flow information. And then we analyse that and find out, maybe, when were the ten worst river flow droughts according to the hydrological model? I’m a bit concerned that the models we have aren’t suitable for doing that in some parts of the UK. … We have 100 years of climate data but not a hundred years of river flow. But I still think there’ll be a lot of value in using the 40 or 50 years that we do have on river flow and trying to use the observations of river flow to understand what’s this connection between climate and river flow—how would we recognize a drought by looking at the climate? (Project scientist)

This quote captures the complexity of defining an environmental phenomenon for the purpose of computer simulation modelling. The scientist talks about the idea of using climate data to drive hydrological models to produce river flow data as model outputs, but also considers the possibility of doing it differently. It would be possible to use the limited number of observations of river flow that are available to identify corresponding climate data. These two approaches start at opposite ends—the first in climate and the second in rivers; however, they both rely on computer simulation modelling and both ways of calculating drought would provide the project with a shared starting point.

There is more to defining drought than deciding which data to use to get started. The climate models used in the project would generate event sets (data that could have been observed if there had been drought) with a resolution of 25 km^2^ per modelled ‘pixel’. This prompted the question of at what resolution the risk of the models missing droughts in smaller catchments that nonetheless could be relevant for hydrological or water quality modelling? How many pixels of low rainfall does it take to simulate drought realistically? It could be important to study local but very severe droughts. This is a question that would not occur to a person studying drought in the field, since the spatial extent of a drought would in that case be a question of comparing observations with a map. However, any map-using field scientist would find that the edges of droughts are not well defined, and this also posed a challenge for the modellers: How should they account, quantitatively, for the blurry boundary between areas in drought and adjacent areas not in drought? To compound the challenges, droughts are not only spatially but also temporally fuzzy; it is very hard to predict when a drought will begin or end. Droughts may, or may not, happen after periods of low rainfall, but the exact point at which the lack of rain turns into drought depends on a multitude of contextual factors. The project scientists appointed a small task force to suggest a definition of drought that all the modelling teams would use.

After much in-depth discussion and analysis, the modellers settled on a three-dimensional definition of drought. The first dimension was based on the Standardized Precipitation Index (SPI) that is ‘widely used to quantify and compare droughts as its simplicity and versatility means that it can be used to characterize a range of drought types using rainfall data alone’ (Internal project note, 2014). This choice enabled the climate modellers to draw the boundary between drought and non-drought in terms of rainfall. Second was a decision on the ‘size’ of droughts that would be addressed in the project. The climate modellers would draw the boundary of a drought, defined according to the SPI, across a spatial extent of a minimum 3 × 3 pixels of 25 km^2^ each. Third was the timing of drought onset, the project modellers choose to use criteria used by the Environment Agency (EA) and the water industry: two winters and one summer with less than average rainfall.

The stakeholder interaction during this phase of the project focussed on building networks, presenting the project at events to which stakeholder representatives were invited, and creating written information to introduce the project to stakeholders with different interests. The scientists did not involve stakeholder representatives directly in their discussions of how to define drought for research purposes, but they selected indicators and parameters widely used by the actors in the drought governance space.

That there was limited involvement of stakeholder representatives in the process that rendered drought an object conducive to computer simulation modelling could be interpreted as a failure to genuinely engage with societal actors (Allison et al., 2018; Malard et al., 2017). This would certainly be the case if the stakeholders invited to participate had been people who could be expected to understand drought differently from the scientists, for example local communities, but as already mentioned the stakeholder representative in the drought project had natural science and engineering backgrounds and used computer models in their own professional practices. In contrast, differences between institutional and lay perspectives on the objects of environmental modelling are highlighted in participatory modelling, which usually aims to include experience-based local knowledge in the computer model representations of objects and processes (Lane et al., 2011; Whatmore & Landström, 2011).

For the institutional stakeholders, the indices and geographical scales selected by the scientists were already firmly established in their modelling practices. In the idiom of co-production, the way in which drought was constructed by the project scientists was integral to the established drought discourse. The drought project rendered drought digital in a way that faithfully reproduced how it was talked about as an environmental phenomenon by the key actors in the UK drought governance space. This digital drought fully agreed with how actors constructed it. And it worked with the innovative modelling relay, contributing to the development of scientific understanding of drought as a continuous process, starting with climate and weather drivers, to catchment processes, to water supply, agricultural and economic impacts. This definition of drought would make it possible to add new scientific knowledge to that which already existed, a cumulative improvement of the scientific representation of drought in the UK. The scientists also expected their digital drought to enable the development of research outputs that the stakeholder could easily apply in their drought management practices.

## Data hunger

From the outset, the scientists in the DWS programme identified data as a key object that would attract scientific and societal interest in the programme. Data holds a crucial role in environmental science computer modelling, and the models require big data both in order to run and to validate outputs. Data is also important in environmental governance; models in water management require data for the same reasons and decision-making draws on data.

Data has always been important in the environmental sciences and digital technologies have made it possible to generate new and more data. Gabrys (2016) argues that environmental big data defines an emerging field, prompted by the monitoring of environmental change on the one hand, and the advances in sensor technology on the other. She understands these as ‘particular ways of materializing environments and ways of acting on environmental problems’ (Gabrys, 2016, p. 2). The push for open data amplifies the idea of data as necessary for all kind of actions, though there are limits to data as a guide to action (Jasanoff, 2017).

The DWS programme’s funding body required that scientific data generated in all four projects were made available to other researchers. Such data sharing amongst scientists has become routine in environmental modelling, according to a scientist in the programme:

[W]e’re happy to have other people looking at our data because before we’re saying it’s mine, it’s only me that can look at it. Now we’re happy to do that which is the first step. (Project scientist)

A driver for data sharing amongst modellers is that computer models require more data than any one research project would be able to generate from observations of environmental processes occurring outside human control, such as droughts. One project scientist explained:

One of the challenges of models, particularly academic models, is that they tend to be rather data hungry. So you have to have all of the data, so it’s much more challenging generally to get the data into a format to drive the model than it is to actually build the model sometimes because the data tend to be quite messy, tend to have gaps, they tend to need to be matched and synchronized in different locations. (Project scientist)

This illustrates the challenges of data in modelling which is often conceived of as an analytical activity, expressing casual relationships in mathematical models and computer code, but in water modelling data is key to generate knowledge and to assess the quality of models.

The drought project relied on data produced by other actors, such as the water utility companies. In the UK the water industry and regulatory bodies generate data that scientific modellers need—the EA is obliged to keep records of various aspects of rivers and lakes, and the water companies collect data to manage their infrastructure systems for water supply and sewage treatment. This is one feature that grants the water companies importance in the drought governance space. According to Lange and Cook (2015, p. 254), water utility companies ‘occupy a central, powerful position in the governance space also because they carry out statutory regulatory functions’, which includes collection of monitoring data. That profit-making businesses play such a key role is an expression of a significant interdependence of private and public actors in the UK drought governance space. The need for scientists to rely on the water industry to access important environmental data is an example of the significant interdependence. That water companies and regulatory agencies generate data needed to model drought provide additional reason for scientists to involve in mutual exchange with these stakeholders. To obtain data from the actors in the drought governance space, the drought project scientists identified and reached out to scientific experts in relevant organizations and negotiated formal data licensing agreements involving the legal departments in universities and businesses.

Even with data from all the relevant actors in the drought governance space, data scarcity remained a challenge for the drought modellers. When looking for patterns and trends scientists need to know that they have representative data that, for example, captures full cycles of environmental processes, as a odeler said:

[I}n order to understand what’s going on, you need to have observations over quite a period because it allows you to put any individual period into context. So, for example, you might want to look at the period 1970 to 1980—if you monitor for that period, it only tells you about that period. So your view of that is completely changed if you have a dataset from 1940 to 2000 … you can see the period that you’re interested in in a wider context and if there’s a 50 year cycle, for example in there, it might appear as a trend in your chosen decade but it’s not actually, it’s just part of the cycle. So there are all of these questions that if I could get any data I wanted, I’d like as much data as possible, I’d like everything monitored every 15 minutes from the beginning of time until now. (Project scientist)

Collecting data for future research has not always been a major concern in drought management, and thus data only exists for a limited historical time. The scientists’ desire for more data—longer time series, better spatial coverage, more phenomena—highlights the driver for big data in model-based environmental research.

### Making drought data

Environmental data is highly heterogeneous, being collected by a variety of devices (Garnett, 2016). In addition, we note that computer modelling creates new data (see, e.g. Edwards, 2010; Leonelli, 2014; Winsberg, 1999). In the model-centred research of the drought project the scientists did not collect new observational data, but they did produce data by modelling at every stage in the relay. Using models to create data is common practice in water modelling, an interviewee doing research on drought independently of the DWS programme explained:

I use river flow data and some of that has gaps in and if I want to compute certain indicators and certain metrics of drought severity I have to fill the gaps in the data. So, I use another model called IHACRES …. So that’s what I’m using to fill gaps and even things like using my long rainfall records to develop long flow records based on the same data. (Environmental modeller)

Recognizing the impossibility of getting observation data of past events, this scientist views the creation of data by modelling as a routine aspect of doing research. Trying to obtain data from the beginning of time, as the interviewee joked in a quote above, appears to be possible when it can be created by models.

The climate modellers in the drought project created hindcast and synthetic data. The hindcasts comprised model-based six-hourly reconstructions of weather that occurred in the past, 1851-2014. This was created using publicly available data generated by the Twentieth Century Reanalysis Project that were made publicly available via a digital platform (see NOAA, 2023). How modellers in the drought project used data generated by others as the starting point for their own production of data complicates Strasser’s and Edwards’ (2017) claim that climate science is the one exception to the limited re-use of open big data. They argue that ‘the vast majority of data preserved is still used only once (if ever), and so far—with important exceptions—even most published data are never reused by anyone other than the original producer’ (Strasser & Edwards, 2017, p. 340). The relay format of the drought project meant that the re-analysis data were incorporated in the event sets used by all the modelling teams to analyse hydrological, ecological and economic processes.

After completing the hindcast, the climate modellers in the drought project went on to produce three synthetic event sets: a baseline comprising drought weather conditions that could have occurred between the years 1900 and 2006, a near future data set with such weather that might occur in the years 2020 to 2050 under the specific IPCC emission scenarios of RCP8.5 and, using the same emission scenarios, a far future with possible weather in 2070–2100.

## Useful data products

The data produced at the different stages of the drought project modelling relay was also curated and made available to other users than scientists. To create data for uses other than scientific research arguably changes its status from shared resource to product offered to customers. The data produced by the modellers in the drought project was disconnected from the origin and packaged as products available for use. In the final report of the DWS programme titled ‘About Drought’, a key ‘data product’:

The UK Drought Portal is a near real-time tool allowing users to explore up-to-date data and monitor current regional dry weather status across the UK. It went live in 2015 based on earlier work and understanding of user requirements for historic drought information. It is focused on standardized drought indicators and enables consistent comparison of different areas regardless of how wet they are. It was used in 2018 as a tool for communicating complex water data comparisons to help decision-makers understand current water resources against drought conditions. (Stevens et al., 2020, p. 44)

The DWS programme also produced a central register of the data sets made available:

The Drought Data Hub provides a simple, visual summary of the huge data outputs from About Drought—and allows users to quickly get access to the data for a specific area or river. It also shows a snapshot of what future flows are going to look like for any of 300 rivers across Great Britain (Northern Ireland data is not currently available). You can view both spatial coverage and detailed information. (Stevens et al., 2020, p. 45)

In addition to the on-line data visualization tools there is a list of the data sets generated by the programme with urls to makes it possible to access them directly.

A broad range of drought governance actors were interested in the data produced by the DWS programme. The drought project stakeholders most interested in the new data were professionals with modelling expertise who worked in technical consultancies, in the water industry and in the regulatory agencies. These experts shared with the project scientists a natural science modelling perspective and in the discussions with stakeholders about the contribution the project would make to drought management it was clear that they considered new data sets to be the most important. The data made available would make it possible for modellers to compare their findings to the scientific analyses. According to one stakeholder representative,

the drought event set will be … an output, at last, that really will have an impact … help consultants and other people for water companies to then model water resources over a much larger, much more subtle set of scenarios to do with water scarcity and drought. (Stakeholder representative)

This sentiment is echoed by professional stakeholder representatives in the other projects, as the DWS programme followed up on the usefulness of the outputs of the four research projects with users of the new data. In the About Drought report there are interviews with stakeholder representatives who talk about the value of the data produced in the programme, one interviewee says: ‘[C]onfidence in evidence data and About Drought’s better and more timely presentation of data is helping decision-makers to better manage uncertainty’ (Trevor Bishop, quoted in Stevens et al., 2020, p. 7).

The About Drought report repeatedly emphasizes the usability of programme outputs and the appreciation of the research among diverse stakeholders. It presents the impact of the DWS programme in a way that bears witness to the effectiveness of stakeholder involvement, and includes numerous testimonies by stakeholders:

A representative for a water utility company says: ‘The Drought Portal gives us a spatial as well as a temporal picture and it confirms our data in a very quick and easy way. We have our own drought severity calculations for single sites but to have it shown for catchments is very useful. The visuals are easy to convey to others in Yorkshire Water and we used some of it to support our drought permit applications to look at the severity and extent of how conditions developed over time.’ (Miranda N, quoted in Stevens et al., 2020, p. 25)

This data represents drought in a way to which both scientists and other actors are committed. There was also interest in the data among NGOs and local environmental groups which demonstrates the penetration of the idea that scientific data is necessary for advocacy. The scientific ambition to make data easily accessible to users outside of scientific institutions also contributes to establishing data as necessary for decision-making. Jasanoff (2017, p. 6) points out that today decisions must be seen to rely on ‘legitimated approaches to producing public facts and public reason’, which in drought governance means approaches grounded in scientific data. Some STS scholars think that scientific numbers offer decision-makers an illusion of certainty ‘at odds with the uncertainty that characterizes many complex and multidimensional policy issues’ (Kovacic, 2018, p. 1040). Rieder and Simon (2016, p. 4) argue that numbers ‘offer a sense of fairness and justice, a way of making decisions without having to decide, a chance to de-politicize legislation’. Notwithstanding such critiques, scientific data and quantitative information continue to attract decision-makers and provide a rationale for institutional and commercial actors to get involved with scientific projects.

## Why no new decision support tools?

While the drought project successfully engaged with stakeholders in the production of new drought data, scientists’ ambition to create model-based decision support tools for use in drought management was less fruitful. The lack of success was not due to lack of interest in modelling in water management. The value of modelling for managing water resources and other water issues has been recognized since the 1970s. As computers became common in the offices of water managers user friendliness, technical stability and reliability were required of models. Over time, software development for use in water management emerged as a distinct field of expertise drawing on knowledge in water science, software engineering and water management.

As computer models used in scientific research on water and modelling software packages used in water management diverged, scientists aiming to contribute to modelling in water management adopted the notion of Decision Support Systems or Tools (DSS/Ts) (McIntosh et al., 2007). This concept positions computer models in a bigger assemblage of tools, knowledge and skills that can underpin decisions about which plan of action to implement or which strategy to employ. The emphasis shifts away from using computer models to generate the kind of representation and predictions in which scientists are interested and towards models contributing to the assessment of potential consequences of management interventions.

Despite a changed understanding of the role of modelling, DSS/Ts developed by environmental scientists have had limited uptake in decision making. MacIntosh et al. (2007, p. 641) identify the most important barrier to the uptake of such tools in policy as the ‘issue of usability by groups other than the developers’. A study of landscape and environmental management found that although ‘a large number of EU research programme funded efforts to bridge the science-practice gap by developing IS/DSS [information and decision support systems] on agricultural and environmental issues, the expected value added in IS/DSS uptake and impact on end-users seem to fall short’ (Zasada et al., 2017, p. 73). As in previous analyses, Zasada et al. (2017) ascribe this failure to a lack of stakeholder involvement: Scientists create models that they imagine could be useful without engaging with actual decision-makers to find out what they really need. This would imply that if scientists pay more attention to what stakeholders need, their DSS/T products will attract more use which has prompted calls for increased stakeholder engagement in model-building water science.

However, as we have noted, stakeholder engagement has been common for decades in this domain, with little effect on the uptake of DSS/T. Alternative understandings are needed.

STS scholars point to the context as decisive. Svetlova and Dirksen (2014, p. 561) explain that in decision-making ‘models are elements of a very particular situation, in which knowledge about the present and the future is limited but dependence of decisions on the future is distinct’. Widening the scope from single organizations to wider societal contexts. Zeiss and Van Egmond (2014, p. 633) examine national policy in the Netherlands, finding that ‘models tend to be adopted when they are constructed in a stable political environment’ and that ‘the level of stability of the scientific realm with respect to both epistemology *and* uncertainty seems decisive for the usefulness of models in policy making processes’. Such contextualizing perspectives are needed to understand the failure of the drought project to generate a new DSS/T.

## Modelling in UK drought management

In the UK water industry, computer modelling is a key approach in both long-term planning and day to day management of water resources. Water companies use scientific data and science-based tools to develop plans that they submit to the regulatory authorities to demonstrate that they are prepared to deal with changing circumstances. All UK water companies are required to develop drought plans that describe what they will do to secure the delivery of water during droughts of varying duration and severity. The responsible Government department, Defra, explains this to the industry:

A drought plan is an operational plan that sets out what actions you will take before, during and after a drought to maintain a secure supply of water. It also sets out how you will assess the effects, including the environmental impacts of your actions and what you will do to monitor and prevent or mitigate these effects. You are expected to fully comply with your drought plan and should expect scrutiny and challenge if this is not the case. (Defra, 2015)

In addition to securing water supply in the event of drought, the water companies must also make sure that their actions do not inflict unacceptable environmental damage. Drought management is complex: It is not enough to figure out how to increase water supply, but the consequences of the solutions must also be monitored and managed. Computer modelling enables assessment of possible consequences of different actions:

We expect you to test your plan using examples. … These could be previous events or modelled design droughts and as a minimum, you should provide a return period/probability, intensity and duration of your chosen events as worked examples. (Defra, 2015)

Although Defra expects water companies to use modelling tools, at the time of the DWS programme the companies mostly relied on rules of thumb and historical data to create their plans:

The scenarios that we use in terms of our drought planning are really pretty simple. We look at average rainfall and then we will look at, say, 80% to 70%, 60%. The lowest that we tend to look at is a 60% scenario, occasionally 50% but it’s very unusual in the historical record that you get more than a month or two with 50% of average rainfall or lower. So that’s why that’s the lowest scenario we use but we also use actual scenarios of historic rainfall. So, we’ll get to a point where we’ll say what happens if we have the 1930/34 rainfall or the 1975/76 rainfall and that gives us an idea of how bad things can get. (Stakeholder interview)

Basing future drought action on knowledge about past droughts was considered unsatisfactory by the scientists in the drought project. They wanted to replace this way of working with a ’risk-based approach’, that relied on more sophisticated modelling and big data, which the scientists considered to be more rigorous. To maximize their chance to push such a change in practice through to the water industry, the scientists planned to release their research outputs when it was time for the five-year drought plan review cycle prescribed by the Government.

The update of drought plans at regular intervals, known in advance, creates a process with clear timelines and predictable opportunities for scientific intervention. However, there is more to the dissemination of models and model-based tools than knowing when to present them to potential users. The rules of thumb used to develop drought plans are only one component in the complex planning and management of water resources. Another important element is the industry standard for models. The actors in the drought governance space who value computer simulation modelling and the information it generates have their own models. The priorities for modellers in water management are that models are easy to use, that they can be applied to local problems without needing to rewrite the programme and that they are established as standard tools among most actors in the governance space.

To understand the ramifications of established modelling approaches, flood risk management provides an illustrative example. Drought modelling is less regulated than the modelling of flood risk, where there are explicit requirements to use certain software packages in the guidelines issued by EA/Defra. Anybody wanting to introduce a new modelling approach in this field is required to demonstrate that it performs at least at the same level as the programmes benchmarked in model comparison exercises, which are undertaken by consortia of scientists in different organizations at regular intervals under the auspices of Defra (Landström, 2023). For drought plans there are no explicit guidelines for model choice. The modellers working in water companies use water industry models, often proprietary software packages developed by the larger, most well-resourced companies. Such models are available for licensing by other companies, which creates a shared perspective on the management of the problems across organizations and geographical areas. The embeddedness of proprietary industry modelling software in economic and regulatory contexts, as well as in established expert practice, makes it very difficult to replace them with new, scientific models.

A scientific article published by one of the projects at the end of the DWS programme provides some clues to why the new modelling approaches were not adopted by stakeholders. Presenting modelling outcomes from their project the authors advocate for water management to adopt scientific modelling approaches that they consider ‘sufficiently mature to form the basis for standard methods for water resources planning’ (Hall et al., 2020, p. 443). They suggest a new way for the water industry to work with models in decision-making in which a ‘preliminary screening of an initial long list will eliminate proposals that are obviously not viable, resulting in a feasible option list which is subject to secondary screening and preliminary studies, which will further eliminate some options, whilst also providing the information on costs, operation rules and reliability that are needed for simulation’ (Hall et al., 2020, p. 445). This would be followed by tests of options in different combinations through modelling. The testing would be done by applying an ensemble of models addressing different aspect of the problems associated with drought. This would, according to the authors, allow the water companies to settle on the most cost-effective and least environmentally damaging strategy to manage droughts.

The models presented in the article are scientific computer programmes, not user-friendly software that could easily be applied to any local system. To become useable tools the models would require significant software development in addition to testing and benchmarking of their representational quality (Wurbs, 1998). The suggestion for how to assess the available drought management options amounts to a major change in the process, making it more like doing scientific research. Perhaps most challenging, the proposal ignores the constraints placed on water utility companies by their relationships with other actors in the drought governance space. The authors seem to think that a water company is free to adopt any new way of working that it would like. Considering the detailed instructions by the regulatory authorities for how to prepare the statutory drought plans, the possibilities for experimenting with new model-based approaches that have only been tried in one scientific project is extremely limited.

Despite three years of stakeholder interaction, the scientists authoring the paper appear not to have grasped the ways in which the relationships between the actors in the drought governance space militate against the adoption of new scientific, untested, model-based ways of working. Drought planning is only one of the responsibilities of water companies and many aspects of it is done in collaboration with other actors, with different priorities, in the drought governance space. To adopt computer modelling programmes directly from one scientific project is not even remotely possible. A new model would have to undergo extensive testing and software development before even being trialled in actual drought planning.

## Discussion: A co-productionist perspective

It is, of course, impossible to provide decisive empirical evidence for why something did not happen, in this case the creation and effective dissemination of model-based tools for decision-making in drought management. However, the STS literature shows that the uptake of model-based tools among users is much more complicated and complex than what scientific modellers imagine. Stakeholder involvement is, in itself, not enough to transfer scientific models into water management decision-making. We can use Jasanoff’s (2004a) four-dimensional idiom of co-production to consider possible reasons for why no successful model-based decision tools emerged in the drought project while the new datasets and data products were created and found favour among stakeholders.

In terms of identities, the drought project did not prompt any changes among those already firmly established across the drought governance space. On the contrary, the identities of individuals and collectives were affirmed by the stakeholder engagement. Stakeholder representatives were individuals with science-based expertise and the authority to speak for their collective in the scientific realm. Hypothetically, new model-based decision-support tools could have undermined existing expert identities if their adoption had changed practices and the knowledge required. However, the focus on data meant that new data could be used with the analytical tools already established, supporting and strengthening existing forms of expertise in the organizations.

In terms of discourses, the scientists and the stakeholder representatives may have talked about drought in the same way but they did not use models for the same purposes. To the scientists, drought was a process that needed to be better understood. In contrast, the water industry talked about drought as a sociotechnical process that had to be managed: Drought plans are about actions and their potential consequences, and the natural process of drought only needs to be understood to the extent that it intersects with the technical system and the measures implemented to ensure the provision of water to customers. Although the scientists used drought indices and definitions that were standard in the drought governance space, modelling plays different roles in scientific and management drought discourses. In comparison, the data sets produced by the scientific programme fitted seamlessly into the drought management discourse.

The scientists aimed to create better representations of droughts in the UK. The water utility companies aimed to prevent, or at least mitigate, the chain of events that makes drought a societal problem. Detailed representation of unmitigated drought, or of all possible mitigation measures, is not necessarily relevant for decision-making. Companies, which have duties and responsibilities to regulators, customers and other actors, need to map out the effects, benefits and risks of the measures at their disposal. There is no point in spending time considering all possible situations when at issue is what is most feasible here and now. Yet they could make use of the new data to, for example, run more sophisticated water management scenarios, without any changes of how droughts are represented.

When it comes to institutions, we see in the drought governance space the importance of the formal and informal relationships between actors in the UK. The space is created through a network, where changes in one relationship could have unintended effects elsewhere; such spaces are appropriate for the current governance format in most environmental domains. The regulatory framework that a government department such as Defra can implement also depends on other actors, such as parliament, water users, riparian owners, and local councils. We found that some relationships between actors in this governance space were formal, mandatory and prescriptive, for example, regulatory frameworks for water industry planning of future activities. Unfortunately for the plan to extend the use of their models to the water industry, the drought scientists did not acknowledge this complex network.

## Conclusion

This paper set out to clarify the limitations of stakeholder involvement as a way for science to generate societal impact. The ambition was to challenge the persistent belief amongst environmental scientists, funding agencies and stakeholder engagement experts that involvement of individuals representing stakeholder organizations will lead to increased uptake of new computational tools in environmental management. From an STS perspective this belief ignores the dynamics of the governance context. By examining a recent model-based drought research programme in the UK, we presented an analysis that contextualized both success and failure with transferring outputs from drought science to stakeholder organizations.

The illumination of the different roles of data and computer models in the relationships between key actors in the drought governance space makes it possible to understand why the data was embraced by water management actors and the models were not. Since environmental data is today regarded as the foundation of all decision-making, drought data generated by modellers were highly valued by actors in the drought governance space.

To maintain trusting relationships in the drought governance space, computer modelling tools need to be standardized. To be approved, modelling tools are turned into maintained software, properly documented and benchmarked. Even if the stakeholder representatives in a research project might find a new modelling tool useful, it takes much more than their appreciation for an organization acting in the drought governance space to adopt a new modelling approach.

That the involvement of stakeholder representatives in a scientific project is not likely to change the way drought is modelled by decision-making bodies and policy actors does not mean that approaches in the drought governance space are static. New models are adopted in drought management, and the software packages set as standards today are not the same (with regard to the underlying scientific models) that were used a decade ago. We leave for further research understandings of the process of renewal of the scientific knowledge (embodied in computer model representation of complex processes) used in drought and environmental management.

## References

[bibr1-03063127231199220] Allison A. E. Dickson M. E. Fisher K. T. Thrush S. F. (2018). Dilemmas of modelling and decision-making in environmental research. Environmental Modelling & Software, 99, 147–155.

[bibr2-03063127231199220] Betsill M. M. Bulkeley H. (2006). Cities and the multilevel governance of global climate change. Global Governance, 12, 141–160.

[bibr3-03063127231199220] Beven K. (2019). How to make advances in hydrological modelling. Hydrology Research, 50(6), 1481–1494.

[bibr4-03063127231199220] Chilvers J. Kearnes M. (2020). Remaking participation in science and democracy. Science, Technology, & Human Values, 45(3), 347–380.

[bibr5-03063127231199220] Department for Environment, Food and Agriculture. (2015). Collection: How to write and publish a drought plan. Retrieved 10 July, 2020, from https://www.gov.uk

[bibr6-03063127231199220] Edwards P. N. (2010). A vast machine: Computer models, climate data, and the politics of global warming. MIT Press

[bibr7-03063127231199220] Gabrys J. (2016). Practicing, materializing and contesting environmental data. Big Data & Society, 3(2). 10.1177/2053951716673391

[bibr8-03063127231199220] Garnett E. (2016). Developing a feeling for error: Practices in monitoring and modelling air pollution data. Big Data & Society, 3(2), 2053951716658061. 10.1177/2053951716658061

[bibr9-03063127231199220] Giordano R. Pluchinotta I. Pagano A. Scrieciu A. Nanu F. (2020). Enhancing nature-based solutions acceptance through stakeholders’ engagement in co-benefits identification and trade-offs analysis. Science of the Total Environment, 713, 136552. 10.1016/j.scitotenv.2020.136552 32019015

[bibr10-03063127231199220] Goodwin D. Raffin M. Jeffrey P. Smith H. M. (2019). Collaboration on risk management: The governance of a non-potable water reuse scheme in London. Journal of Hydrology, 573, 1087–1095. 10.1016/j.jhydrol.2017.07.020

[bibr11-03063127231199220] Hall J. W. Mortazavi-Naeini M. Borgomeo E. Baker B. Gavin H. Gough M. Harou J. J. Hunt D. Lambert C. Piper B. Richardson N. (2020). Risk-based water resources planning in practice: A blueprint for the water industry in England. Water and Environment Journal, 34(3), 441–454.

[bibr12-03063127231199220] Hamilton S. H. Pollino C. A. Stratford D. S. Fu B. Jakeman A. J. (2022). Fit-for-purpose environmental modeling: Targeting the intersection of usability, reliability and feasibility. Environmental Modelling and Software, 148, 105728. 10.1016/j.envsoft.2021.105278

[bibr13-03063127231199220] Jasanoff S. (2004a). The idiom of co-production In Jasanoff S. (Ed.), States of knowledge: The co-production of science and social order (pp. 1–12). Routledge.

[bibr14-03063127231199220] Jasanoff S. (2004b). Ordering knowledge, ordering society. In Jasanoff S. (Ed.), States of knowledge: The co-production of science and social order (pp. 13–45). Routledge.

[bibr15-03063127231199220] Jasanoff S. (2017). Virtual, visible, and actionable: Data assemblages and the sightlines of justice. Big Data & Society, 4(2), 205395171772447. 10.1177/2053951717724477

[bibr16-03063127231199220] Jaton F. (2017). We get the algorithms of our ground truths: Designing referential databases in digital image processing. Social Studies of Science, 47(6), 811–840. 28950802 10.1177/0306312717730428 PMC5697567

[bibr17-03063127231199220] Jensen C. B. (2020). A flood of models: Mekong ecologies of comparison. Social Studies of Science, 50(1), 76–93. 10.1177/0306312719871616 31537152

[bibr18-03063127231199220] Kovacic Z. (2018). Conceptualizing numbers at the science-policy interface. Science, Technology & Human Values, 43(6), 1039–1065.

[bibr19-03063127231199220] Laborde S. (2015). Environmental research from here and there: Numerical modelling labs as heterotopias. Environment and Planning D: Society and Space, 33, 265–280.

[bibr20-03063127231199220] Lahsen M. (2005). Seductive simulations? Uncertainty distribution around climate models. Social Studies of Science, 35(6), 895–922.

[bibr21-03063127231199220] Landström C. (2020). Environmental participation. Practices engaging the public with science and governance. Palgrave MacMillan. 10.1007/978-3-030-33043-9.

[bibr22-03063127231199220] Landström C. (2023). Why won’t water managers use new scientific computer models? The co-production of a perceived science-practice gap. TATuP—Journal for Technology Assessment in Theory and Practice, 32(1), 36–42.

[bibr23-03063127231199220] Lane S. N. Odoni N. A. Landström C. Whatmore S.J. Ward N. Bradley S. (2011). Doing flood risk science differently: An experiment in radical scientific method. Transactions of the Institute of British Geographers, 36(1), 15–36.

[bibr24-03063127231199220] Lange B. Cook C. (2015). Mapping a developing governance space: Managing drought in the UK. Current Legal Problems, 68, 229–266.

[bibr25-03063127231199220] Latour B. (1987). Science in action. How to follow scientists and engineer through society. Harvard University Press

[bibr26-03063127231199220] Leonelli S. (2014). What difference does quantity make? On the epistemology of Big Data in biology. Big Data & Society, 1(1). 10.1177/2053951714534395 PMC4340542 25729586

[bibr27-03063127231199220] Malard J. J. Inam A. Hassanzadeh E. Adamowski J. Tuy H. A. Melgar-Quiñonez H. (2017). Development of a software tool for rapid, reproducible, and stakeholder-friendly dynamic coupling of system dynamics and physically-based models. Environmental Modelling & Software, 96, 410–420.

[bibr28-03063127231199220] Mauz I. Granjou C. (2013). A new border zone in science. Collaboration and tensions between modelling ecologists and field naturalists. Science as Culture, 22(3), 314–343.

[bibr29-03063127231199220] Mayernick M. S. (2019). Metadata accounts: Achieving data and evidence in scientific research. Social Studies of Science, 49(5), 732–757. 31354073 10.1177/0306312719863494 PMC7323761

[bibr30-03063127231199220] McIntosh B. S. Seaton R. A. F. Jeffrey P. (2007). Tools to think with? Towards understanding the use of computer-based support tools in policy relevant research. Environmental Modelling & Software, 22, 640–648.

[bibr31-03063127231199220] McLellan T. (2021). Impact, theory of change, and the horizons of scientific practice. Social Studies of Science, 51(1), 100–120. 32842910 10.1177/0306312720950830

[bibr32-03063127231199220] Morrison M. Morgan M. S. (1999). Models as mediating instruments. In Morgan M. S. Morrison M. (Eds.), Models as mediators. Perspectives on natural and social science (pp. 10–37). Cambridge University Press.

[bibr33-03063127231199220] National Oceanic and Atmospheric Administration. (2023). The twentieth century reanalysis project. Retrieved August 12, 2023, from https://www.psl.noaa.gov/data/20thC_Rean/

[bibr34-03063127231199220] Oliver K. Boaz A. (2019) Transforming evidence for policy and practice: Creating space for new conversations. Palgrave Communications, 5, 60. 10.1057/s41599-019-0266-1

[bibr35-03063127231199220] Parrott L. (2017). The modelling spiral for solving ‘wicked’ environmental problems: Guidance for stakeholder involvement and collaborative model development. Methods in Ecology and Evolution, 8(8), 1005–1011.

[bibr36-03063127231199220] Rieder G. Simon J. (2016). Datatrust: Or, the political quest for numerical evidence and the epistemologies of Big Data. Big Data & Society, 3(1). 10.1177/2053951716649398

[bibr37-03063127231199220] Sandink D. Simonovic S. P. Schardong A. Srivastav R. (2016). A decision support system for updating and incorporating climate change impacts into rainfall intensity-duration-frequency curves: Review of the stakeholder involvement process. Environmental Modelling & Software, 84, 193–209.

[bibr38-03063127231199220] Sismondo S. (1999). Editor’s introduction: Models, simulations and their objects. Science in Context, 12(2), 247–260.

[bibr39-03063127231199220] Smallman M. (2020). ‘Nothing to do with the science’: How an elite sociotechnical imaginary cements policy resistance to public perspectives on science and technology through the machinery of government. Social Studies of Science, 50(4), 589–608. 10.1177/0306312719879768 31603380

[bibr40-03063127231199220] Stevens S. Turner S. Sarkar S. (2020). About drought handbook. Outputs and impacts. The UK Drought and Water Scarcity Programme. UKRI

[bibr41-03063127231199220] Strasser B. J. Edwards P. N. (2017). Big Data is the answer ... but what is the question? Osiris, 32, 328–345.

[bibr42-03063127231199220] Sundberg M. (2006). Credulous modellers and suspicious experimentalists? Comparison of model output and data in meteorological simulation modelling. Science Studies, 19(1), 52–68.

[bibr43-03063127231199220] Sundberg M. (2009). The everyday world of simulation modeling. The development of parameterizations in meteorology. Science, Technology & Human Values, 34(2), 162–181.

[bibr44-03063127231199220] Svetlova E. Dirksen V. (2014). Models at work—Models in decision making. Science in Context, 27, 561–577. 25549444 10.1017/s0269889714000209

[bibr45-03063127231199220] Whatmore S. J. Landström C. (2011). Flood-apprentices: An exercise in making things public. Economy & Society, 40(4), 582–610.

[bibr46-03063127231199220] Winsberg E. (1999). Sanctioning models: The epistemology of simulation. Science in Context, 12, 275–292. 10.1017/S0269889700003422

[bibr47-03063127231199220] Wu W. Maier H. R. Dandy G. C. Leonard R. Bellette K. Cuddy S. Maheepala S. (2016). Including stakeholder input in formulating and solving real-world optimization problems: Generic framework and case study. Environmental Modelling & Software, 79, 197–213.

[bibr48-03063127231199220] Wurbs R. A. (1998). Dissemination of generalized water resources models in the United States. Water International, 23(3), 190–198.

[bibr49-03063127231199220] Wynne B. (2006). Public engagement as a means of restoring public trust in science—hitting the notes, but missing the music? Public Health Genomics, 9(3), 211–220. 10.1159/000092659 16741352

[bibr50-03063127231199220] Zasada I. Piorr A. Novo P. Villaneuva A. J. Valanszki I. (2017). What do we know about decision support systems for landscape and environmental management? A review and expert survey within EU research projects. Environmental Modelling & Software, 98, 63–74.

[bibr51-03063127231199220] Zeiss R. Van Egmond S. (2014). Dissolving decision making? Models and their roles in decision-making processes and policy at large. Science in Context, 27, 631–657. 25549446 10.1017/s0269889714000234

